# Molecular Characterization of Microtubule Affinity-Regulating Kinase4 from *Sus scrofa* and Promotion of Lipogenesis in Primary Porcine Placental Trophoblasts

**DOI:** 10.3390/ijms20051206

**Published:** 2019-03-09

**Authors:** Liang Tian, Aiyou Wen, Shusheng Dong, Peishi Yan

**Affiliations:** 1 College of Animal Science and Technology, Nanjing Agricultural University, Nanjing 210095, China; 2014105052@njau.edu.cn (S.D.); yanps@njau.edu.cn (P.Y.); 2 College of Animal Science, Anhui Science and Technology University, Fengyang 233100, China; aywen2008@126.com

**Keywords:** MARK4, pig, lipogenesis, placenta, WNT, molecular cloning, PPARγ

## Abstract

This study aimed to characterize the full-length cDNA of MARK4 in *Sus scrofa*, and evaluated its potential role in the regulation of lipid accumulation in pig placental trophoblasts and analyzed signaling pathways involved, thereby providing insights into mechanisms for placental lipotoxicity induced by excessive back-fat during pregnancy of sows. The cDNA obtained with 5′ and 3′ RACE amplification covered 3216 bp with an open reading frame of 2259 bp encoding 752 amino acids. Multiple alignments and phylogenetic analysis revealed MARK4 protein of *Sus scrofa* had a high homology (95%–99%) to that of other higher vertebrates. After transfection, enhanced MARK4 significantly promoted lipogenesis in pig trophoblasts, as evidenced by accelerated lipid accumulation and consistently increased mRNA expressions of lipogenic genes DGAT1, LPIN1, LPIN3, LPL, PPARδ and SREBP-1c. Meanwhile, PPARγ remarkably inhibited the stimulating effect of MARK4 on non-receptor-mediated lipid accumulation in trophoblasts. Further analyses revealed WNT signaling enhanced lipid accumulation and activation of MARK4 in pig trophoblast cells. Finally, we demonstrated that WNT/β-catenin signal pathway is involved in MARK4 activated lipogenesis. These results suggest that MARK4 promotes lipid accumulation in porcine placental trophoblasts and can be considered as a potential regulator of lipotoxicity associated with maternal obesity in the pig placenta.

## 1. Introduction

Obese pregnancy has been demonstrated to provoke an adverse intrauterine milieu, and as a result, poor pregnancy outcomes in human beings and some animal species, such as pig [1,2]. Although the connection between fetal development and maternal obesity is confirmed, the underlying mechanisms connecting adverse maternal environment to the fetus remain elusive. As the interface between the fetus and maternal environment, the placenta has become an important source of pathogenic factors affecting fetal metabolism and development [3,4]. Recently, several studies suggested that maternal obesity during pregnancy is associated with elevated maternal circulating levels of fatty acids and inflammatory cytokines, resulting in a lipotoxic milieu within the placenta characterized by increased placental lipid, inflammation and oxidative stress [5,6,7]. Lipotoxicity has been demonstrated to induce placental dysfunction evidenced by maternal obesity associated dysregulation of lipid transport and metabolism in the human or pig full-term placenta [8,9]. Recent evidences further revealed that maternal obesity contributes to decreased placental efficiency (a ratio of fetal weight to placental weight) and excessive placental fat accumulation through an aberrant activation of WNT signaling and PPARδ in placenta from an obesity-prone rat model [10], thus leading to compromised fetal development. Furthermore, our studies showed that WNT signaling and inflammatory NF-κB and JNK signaling are activated in term placenta from sows with excessive back-fat [11], suggesting that maternal obesity may induce lipotoxicity in the full-term porcine placenta. However, the precise cellular and molecular mechanisms responsible for maternal obesity associated lipid accumulation in the pig placenta are still barely understood.

Microtubule affinity-regulating kinase 4 (MARK4) is a member of the AMP-activated protein kinase (AMPK)-related family of kinases, which has been reported to expressed in multiple tissues [12]. As the mammalian homologs of nematode Par-1, microtubule affinity regulatory kinases (MARKs) family contains four members, MARK1(Par-1c), MARK2(Par-1b/EMK1), MARK3(Par-1a/C-TAK1) and MARK4 (Par-1d/MARKL-1), and they share a highly conserved structure consisting of three distinct domains: a catalytic kinase domain, a ubiquitin-associated domain and a kinase associated domain [13]. Studies have implicated Mark4 in diverse physiological processes, including regulation of programmed cell death [14], cell proliferation [15], and glucose homeostasis and energy metabolism [16]. Recent evidences demonstrate that MARK4 promotes adipogenesis and triggers adipocytes apoptosis along with increased adipose inflammation and oxidative stress [17,18]. Moreover, our findings indicated that excessive back-fat is associated with increased activation of MARK4 in pig term placenta, suggesting a potential mechanism for increased activation of JNK mediated mitochondrial apoptotic pathway [19]. All these findings suggest that MARK4 is a versatile protein involved in large number of metabolic processes. However, the regulatory role of MARK4 on placental lipid accumulation, especially in maternal obese condition, is still unknown in porcine. To date, MARK4 gene has been characterized molecularly in several vertebrate species, including pigs [13,20], while the knowledge of molecular structure of MARK4 in *Sus scrofa* (Pig) is still limited, as warrants further studies. 

Given the regulatory role played by MARK4 in adipogenesis and energy metabolism, we aimed to evaluate whether MARK4 expression is correlated with lipid accumulation in pig placental trophoblast cells *in vitro*. In addition, we cloned the full-length cDNA of the MARK4 gene from the placenta of porcine using 5′ and 3′ RACE amplification and employed bioinformatics analysis to identify the molecular characterization and structure of MARK4 from *Sus scrofa*. In this study, we demonstrated that, through activating the WNT/β-catenin and inhibiting the PPARγ pathways, MARK4 promoted lipogenesis in pig placental trophoblasts, implicating MARK4 as a potential regulator of lipid accumulation associated with maternal obesity in the pig placenta. 

## 2. Results

### 2.1. Molecular Characterization of MARK4 Gene

After performing core fragment amplification and 5′ and 3′ RACE, the full-length cDNA of MARK4 gene (GenBank accession number: MH926032) from *Sus scrofa* was obtained (Figure S1). The full-length cDNA covered 3216 bp with an ORF of 2259 bp encoding 752 amino acids. The MARK4 protein had a calculated molecular weight (Mw) of 82535.70 Da and isoelectric point (PI) of 9.70. This amino acid (AA) sequence contained several conserved functional sites, including one proton acceptor (Asp181), one protein kinase ATP-binding region signature (IIe65-Lys88), one serine/threonine protein kinase active-site signature (IIe177-Leu189) and one protein kinase domain (Tyr59-IIe310). Based on the results predicted by the online SABLE program, the secondary structure of this MARK4 protein consisted of 13 α-helices, 13 β-strands and 26 coils (Figure S2).

**Figure 1:**
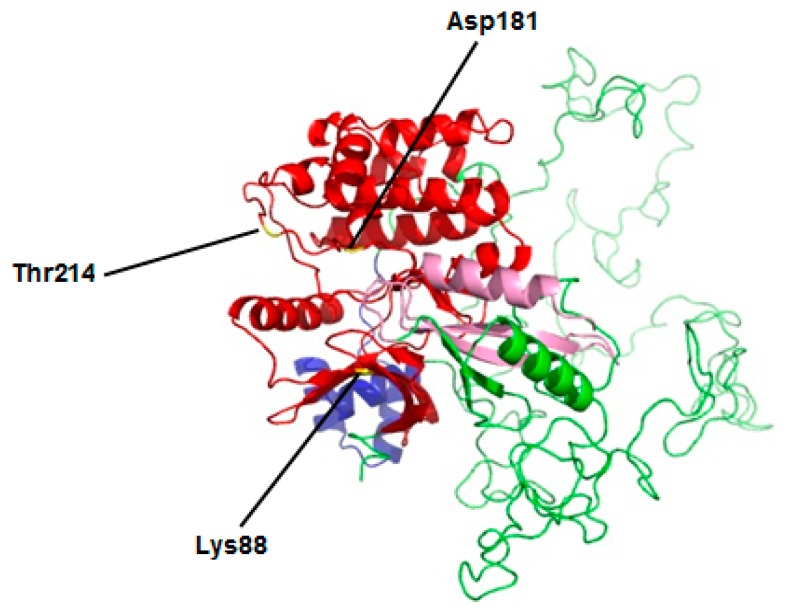
The tertiary protein structures of MARK4 protein in Pig (*Sus scrofa*) modeled by the ProModII program. Kinase domain (KD) colored red, ubiquitin-associated domain (UBA) is blue and kinase associated domain1 (KA1) in pink.

Additionally, conserved motifs were identified in the amino acid sequence of the MARK4 protein, including the activation loop, the catalytic kinase domain (KD), the ubiquitin-associated domain (UBA), the kinase associated domain1 (KA1) and three conserved functional sites (lysine 88 ATP binding site, aspartic 181 active site and threonine 214 phosphorylation site; Figure 1). This MARK4 protein sequence had a high similarity, and showed similar structural features to the MARK4 protein of other species (Figure S3).

### 2.2. Phylogenetic Analysis

The phylogenetic tree among 11 species based on the amino acid (AA) sequences of MARK4 protein was presented in Figure S4. MARK4 of pig (*Sus scrofa*) showed a close phylogenetic relationship with that of human (*Homo sapiens*) and chimpanzee (*Pan troglodytes*). Conservation of MARK4 was also evident from similarity comparisons in NCBI, as the MARK4 protein of *Sus scrofa* showed a high identity (95%–99%) to that of David’s myotis (*Myotis davidii*), Chimpanzee (*Pan troglodytes*), American beaver (*Castor canadensis*), Domestic guinea pig (*Cavia porcellus*), Norway rat (*Rattus norvegicus*), House mouse (*Mus musculus*), Dingo (*Canis lupus dingo*), Horse (*Equus caballus*) and Human (*Homo sapiens*).

### 2.3. MARK4 Increases Lipid Droplet Accumulation in Pig Placental Trophoblast Cells

**Figure 2:**
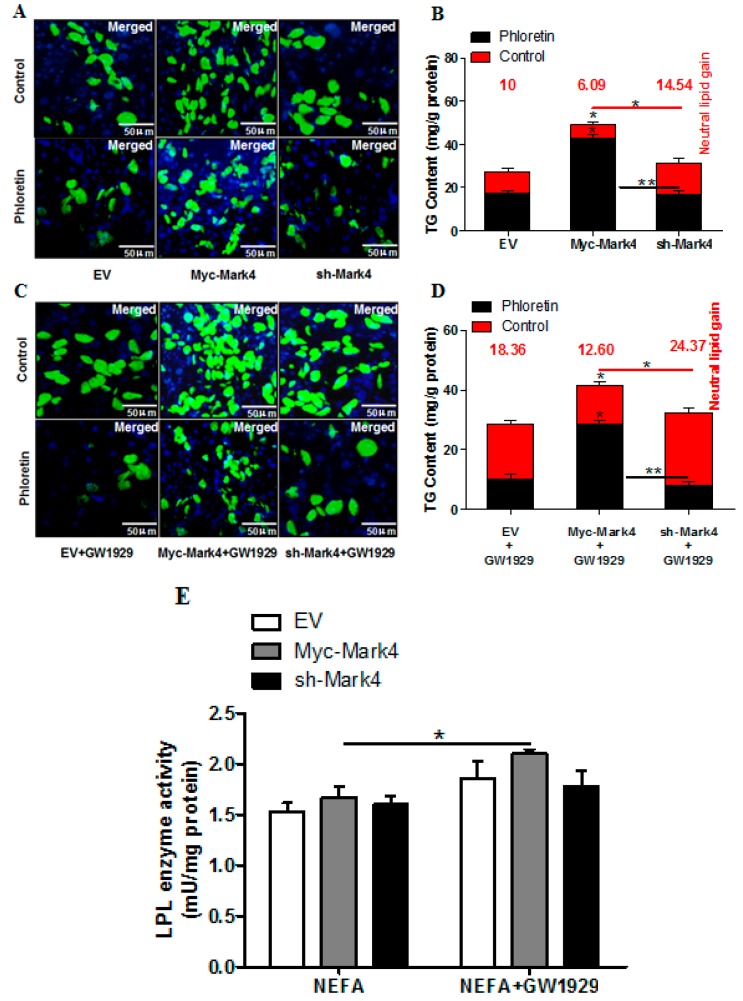
MARK4 promotes lipid accumulation in pig primary trophoblast cells challenged with 400 μM NEFA. (A and C) Representative images (100×) of Bodipy staining after transfection with Myc-MARK4, sh-MARK4 for 48 h in primary (trophoblast cells) isolated from pig placentas. Primary trophoblasts were then incubated with 400 μM NEFA, 2 μM GW1929 or 500 μM phloretin for 24 h (*n* = 3). (**B** and **D**) Quantification of corresponding triglyceride (TG) in (**A**) and (**C**) by ELISA analysis (*n* = 3). The values in red indicate receptor (transport proteins)-mediated fatty acid accumulation by subtracting the values in the presence of phloretin from those in the absence of phloretin. (E) LPL activity (mU/mg protein) after transfection with Myc-MARK4, sh-MARK4 for 48 h in pig primary trophoblasts. Cells were then treated with 400 μM NEFA or 2 μM GW1929 for 24 h (*n* = 3). Values are expressed as mean ± SEM. ** *p* < 0.01; * *p* < 0.05 compared with the control group. Myc-MARK4 group: overexpression of MARK4 group, sh-MARK4 group: knock down of MARK4 group, Control: empty vector (EV) group.

In this study, we speculated that MARK4 could modulate lipid accumulation in porcine placental trophoblast cells. To validate our hypothesis, we initially tested whether overexpression of MARK4 influences the accumulation of fatty acid in cultured term primary pig trophoblasts exposed to 400 μM FA. The results of Bodipy 493/503 fluorescence staining and TG content assay indicated that lipid droplet accumulation was significantly increased in trophoblasts from the Myc- MARK4 group compared with the sh- MARK4 or vector control groups (*p* < 0.05; control panel in Figure 2A,B). 

We next examined whether MARK4 affected receptor (transport proteins)-mediated fatty acid accumulation in cultured trophoblast cells. As shown in Figure 2B, sh-MARK4 treatment increased receptor-mediated fatty acid accumulation in trophoblasts compared with Myc-MARK4 group following 24 h exposure to FA (sh-MARK4: 14.54 ± 2.41 mg/g versus Myc-MARK4: 6.09 ± 1.61 mg/g, *p* < 0.05). Previous studies have shown that PPARγ is involved in regulating fatty acid transport and accumulation in primary human placental trophoblasts [21]. We therefore hypothesized that activation of PPARγ might increase the accumulation of fatty acid in cultured pig placental trophoblast cells. To test this hypothesis, we incubated trophoblasts in the presence or absence of PPARγ-specific agonist GW1929. As shown in Figure 2B,D, activation of PPARγ promoted receptor-mediated fatty acid accumulation in sh-MARK4 treatment following 24 h exposure to FA (sh-MARK4+GW1929: 24.37 ± 1.39 mg/g versus sh-MARK4: 14.54 ± 2.41 mg/g, *p* < 0.05), whereas non- receptor-mediated fatty acid accumulation was significantly decreased in Myc-MARK4 group following GW1929 + phloretin treatment (Myc-MARK4+GW1929: 28.75 ± 1.03 mg/g versus Myc-MARK4: 42.87 ± 1.89 mg/g, *p* < 0.05). In accord with increased receptor-mediated fatty acid accumulation in Myc-MARK4+GW1929 group (Myc-MARK4+GW1929: 12.60 ± 1.22 mg/g versus Myc-MARK4: 6.09 ± 1.61 mg/g, *p* < 0.05), the LPL activity in Myc-MARK4 + GW1929 group was markedly higher than that in Myc-MARK4 group (*p* < 0.05; Figure 2E).

### 2.4. Effect of MARK4 on Key Factors of Lipid Metabolism in Pig Placental Trophoblasts

**Figure 3:**
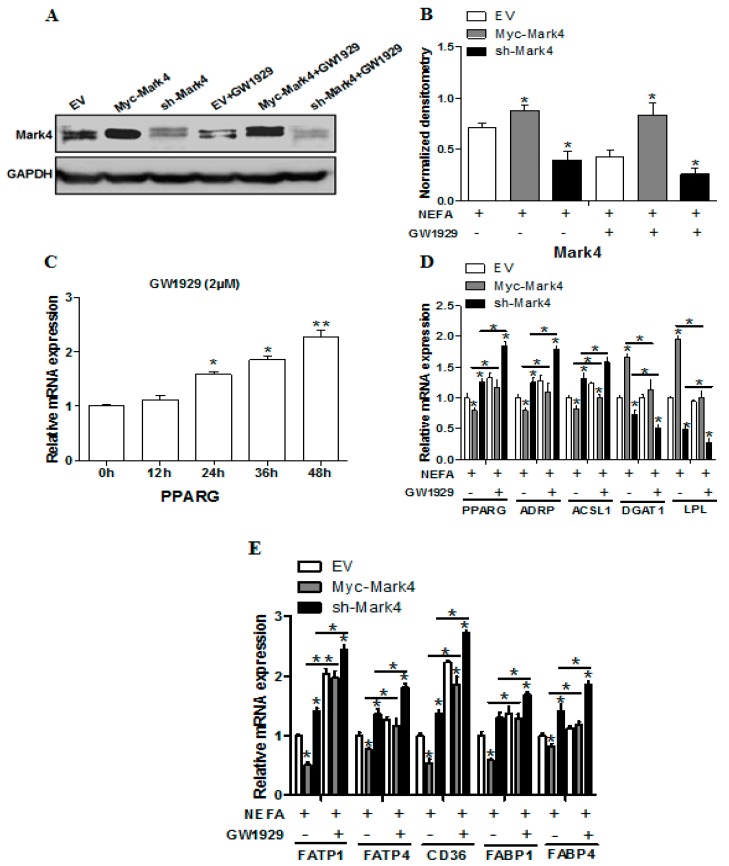
Effects of MARK4 on key molecules of lipid metabolism in pig primary trophoblast cells. (**A**–**B**) Representative immunoblots and densitometric quantification for MARK4 after transfection with Myc-MARK4, sh-MARK4 for 48 h in primary trophoblast cells isolated from pig placentas. Cells were then incubated with 400 μM NEFA or 2 μM GW1929 for 24 h (*n* = 3). (**C**) Primary trophoblasts were cultured and incubated for 0 h, 12 h, 24 h, 36 h and 48 h in the presence of 2 μM GW1929. Relative mRNA expression of PPARγ was detected (*n* = 3). (**D**–**E**) Relative mRNA expression of lipid metabolism-related genes (**D**) and fatty acid (FA) transporters (**E**) after transfection with Myc-MARK4, sh-MARK4 for 48 h in primary (trophoblast cells). Cells were then treated with 400 μM NEFA or 2 μM GW1929 for 24 h (*n* = 3). Values are expressed as mean ± SEM. ** *p* < 0.01; * *p* < 0.05 compared with the control group. Myc-MARK4 group: over expression of MARK4 group, sh-MARK4 group: knock down of MARK4 group, Control: empty vector (EV) group.

We first determined the overexpression of MARK4 by testing protein content of MARK4 gene following transfection and FA treatment. As shown in Figure 3A,B, MARK4 protein increased in Myc-MARK4 group, while sh-MARK4 treatment reduced MARK4 protein (*p* < 0.05). Consistent with increased lipid droplet accumulation following FA treatment, the mRNA expression of genes associated with fatty acid uptake and accumulation, including LPL and DGAT1, was significantly increased in Myc-MARK4 group, whereas the mRNA content of lipid metabolism-related genes, including PPARG (PPARγ), ADRP and ACSL1, was reduced in Myc-MARK4 group compared with the sh-MARK4 or vector control groups (*p* < 0.05; Figure 3D). GW1929, the potent and specific agonist of PPARγ (Figure 3C), was used to examine the regulatory role of PPARγ on MARK4-induced increases in lipid accumulation of trophoblasts. As shown in Figure 3D, GW1929 promoted the mRNA expression of PPARG, ADRP and ACSL1 in Myc-MARK4 group, but the mRNA content of LPL and DGAT1 was decreased in Myc-MARK4+ GW1929 treatment (*p* < 0.05). In accordance with elevated receptor-mediated fatty acid accumulation following GW1929 + sh- MARK4 treatment, GW1929 increased the mRNA content of several fatty acid transporters, including FATP1, FATP4, CD36, FABP1 and FABP4, in sh-MARK4 group (*p* < 0.05; Figure 3E).

### 2.5. WNT Signaling Promotes Lipid Accumulation and Activation of MARK4 in Pig Trophoblasts

**Figure 4:**
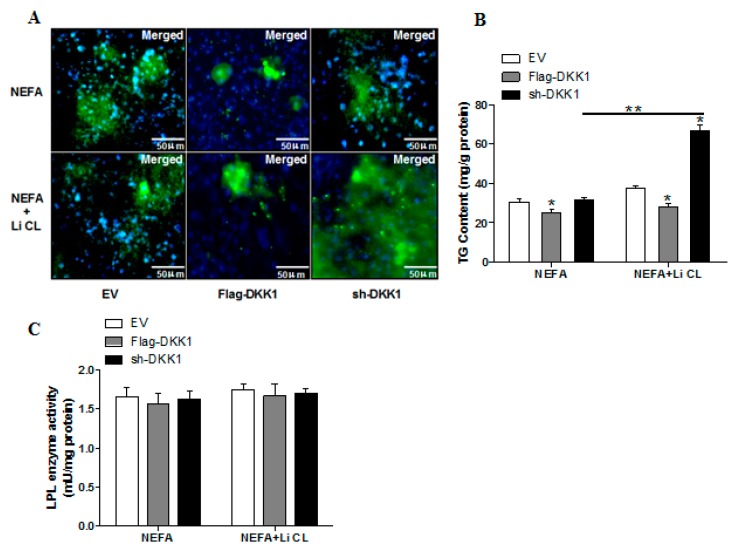
Activation of the Wnt/β-catenin pathway promotes lipid accumulation in pig primary trophoblast cells challenged with 400 μM NEFA. (**A**) Representative images (100×) of Bodipy staining after transfection with Flag-DKK1, sh-DKK1 for 48 h in primary trophoblast cells isolated from pig placentas. Cells were then incubated with 400 μM NEFA or 20 μM Li CL for 24 h (*n* = 3). (**B**) Quantification of corresponding triglyceride (TG) in (**A**) by ELISA analysis (*n* = 3). (**C**) LPL activity (mU/mg protein) after transfection with Flag-DKK1, sh-DKK1for 48 h in pig primary trophoblasts. Cells were then treated with400 μM NEFA or 20 μM Li CL for 24 h (*n* = 3). Values are expressed as mean ± SEM. ** *p* < 0.01; * *p* < 0.05 compared with the control group. Flag-DKK1 group: over expression of DKK1 group, sh-DKK1 group: knock down of DKK1 group, Control: empty vector (EV) group.

Previous experiments in our laboratory and others have shown that an aberrant activation of WNT signaling contributes to significant placental lipid accumulation in obese model of rat or pig [10,11]. In order to further reveal the mechanisms responsible for the increased placental lipid accumulation induced by WNT signaling, we first performed Bodipy fluorescence staining to evaluate lipid droplet accumulation in pig trophoblasts from three groups: Flag-DKK1, sh-DKK1 and Vector control. DKK1 (dickkopf family protein1) is an inhibitor of the canonical WNT signaling pathway [22]. As shown in Figure 4A,B, Flag-DKK1 treatment reduced lipid droplet accumulation in trophoblasts following 24 h exposure to FA (*p* < 0.05), whereas activation of WNT signaling by GSK3β inhibitor LiCL, which was the downstream of DKK1 and blocked the phosphorylation of β-catenin and subsequent proteolytic degradation, significantly increased lipid accumulation in sh-DKK1 treatment (*p* < 0.01). The LPL activity was not affected by Flag-DKK1 or sh-DKK1 treatment in the presence or absence of LiCL (Figure 4C).

**Figure 5:**
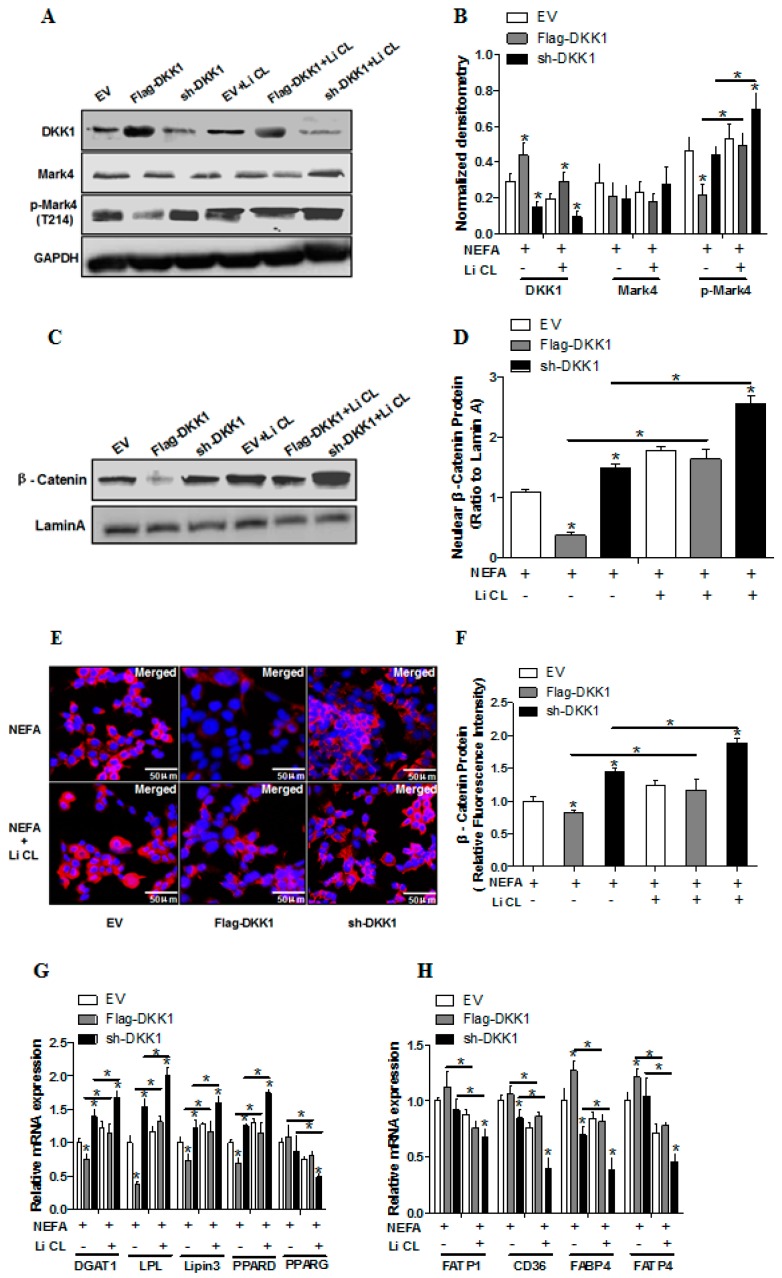
Inhibition of the Wnt/β-catenin pathway blocks key molecules of lipid metabolism and activation of MARK4 in pig primary trophoblast cells. (**A**–**D**) Representative immunoblots and densitometric quantification for p-MARK4 (T214), DKK1 and β-catenin after transfection with Flag-DKK1, sh-DKK1 for 48 h in primary trophoblast cells isolated from pig placentas. Cells were then incubated with 400 μM NEFA or 20 μM Li CL for 24 h (*n* = 3). (**E**) Representative images (100×) of β-catenin immunofluorescent staining after transfection with Flag-DKK1, sh-DKK1 for 48 h in pig primary trophoblast cells. Cells were then incubated with 400 μM NEFA or 20 μM Li CL for 24 h (*n* = 3). (**F**) Quantification of red fluorescence intensity in (**E**) relative to control group (*n* = 3). (**G**–**H**) Relative mRNA expression of lipid metabolism-related genes (**G**) and fatty acid (FA) transporters (**H**) after transfection with Flag-DKK1, sh-DKK1 for 48 h in primary trophoblast cells. Cells were then treated with 400 μM NEFA or 20 μM Li CL for 24 h (*n* = 3). Values are expressed as mean ± SEM. * *p* < 0.05 compared with the control group. Flag-DKK1 group: overexpression of DKK1 group, sh-DKK1 group: knock down of DKK1 group, Control: empty vector (EV) group.

We next determined whether inhibition of WNT signaling affected lipid metabolism in pig placental trophoblasts. Not surprisingly, overexpression of DKK1 increased DKK1 protein content (*p* < 0.05; Figure 5A,B) and reduced β-catenin protein expression within the nucleus (Figure 5C). Notably, LiCL treatment prevented DKK1-induced degradation of β-catenin (*p* < 0.05; Figure 5D); this result was also confirmed by immunofluorescence assay for β-catenin (*p* < 0.05; Figure 5E,F). Consistent with elevated lipid accumulation in sh-DKK1 group following exposure to FA + LiCL, the mRNA expression of genes associated with TG synthesis, including DGAT1, LPL, LPIN3 and PPARδ, were higher in sh-DKK1 + LiCL treatment (*p* < 0.05; Figure 5G), while LiCL treatment reduced the mRNA content of fatty acid transport -related genes, including PPARγ, FATP1, FATP4, CD36 and FABP4, in Flag-DKK1 or sh-DKK1 group (*p* < 0.05; Figure 5G,H). Moreover, phos- MARK4(Thr214) was decreased in Flag-DKK1 compared with the sh-DKK1 or vector control groups, but increased activation of Mark4 was observed in Flag-DKK1+ LiCL treatment (*p* < 0.05; Figure 5A,B). 

### 2.6. WNT/β-Catenin Signal is Essential for MARK4 Activated Lipogenesis in Pig Trophoblast Cells

**Figure 6:**
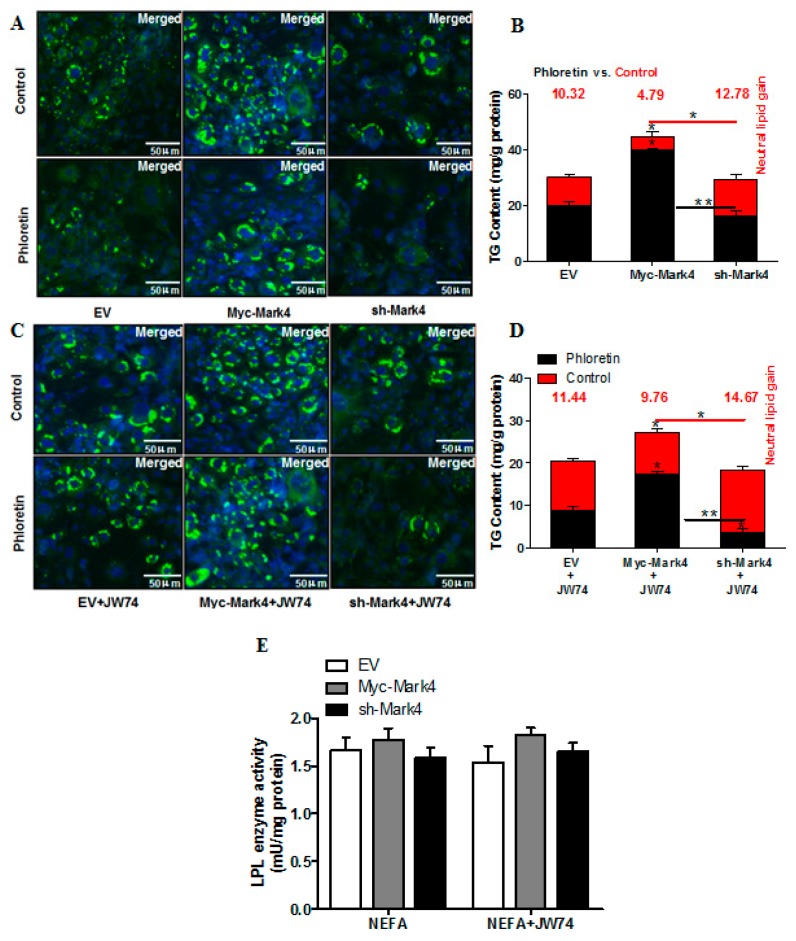
Activation of the WNT/β-catenin pathway by MARK4 promotes lipid accumulation in pig primary trophoblast cells challenged with 400 μM NEFA. (**A** and **C**) Representative images (100×) of Bodipy staining after transfection with Myc-MARK4, sh-MARK4 for 48 h in primary (trophoblast cells) isolated from pig placentas. Cells were then incubated with 400 μM NEFA, 10 μM JW74 or 500 μM phloretin for 24 h (*n* = 3). (**B** and **D**) Quantification of corresponding triglyceride (TG) in (**A**) and (**C**) by ELISA analysis (*n* = 3). The values in red indicate receptor (transport proteins)-mediated fatty acid accumulation by subtracting the values in the presence of phloretin from those in the absence of phloretin. (**E**) LPL activity (mU/mg protein) after transfection with Myc-MARK4, sh-MARK4 for 48 h in pig primary trophoblasts. Cells were then treated with 400 μM NEFA or 10 μM JW74 for 24 h (*n* = 3). Values are expressed as mean ± SEM. ** *p* < 0.01; * *p* < 0.05 compared with the control group. Myc-MARK4 group: overexpression of MARK4 group, sh-MARK4 group: knock down of MARK4 group, Control: empty vector (EV) group.

Having determined that WNT signaling enhanced the accumulation of fatty acids and activation of MARK4 in pig placental trophoblast cells, we next addressed whether WNT/β-catenin pathway was involved in Mark4-induced lipid accumulation in pig trophoblasts. To test this hypothesis, we incubated trophoblasts in the presence or absence of WNT signaling pathway specific inhibitor JW74. As shown in Figure 6B,D, inhibition of WNT/β-catenin signaling by JW74 reduced non- receptor-mediated fatty acid accumulation in sh-MARK4 group following 24 h exposure to FA + phloretin (sh-MARK4+JW74: 3.56 ± 0.80 mg/g versus sh-MARK4: 16.47 ± 1.61 mg/g, *p* < 0.05), whereas receptor-mediated fatty acid accumulation was significantly increased in Myc-MARK4 group following JW74 treatment (Myc-MARK4+ JW74: 9.76 ± 0.90 mg/g versus Myc-MARK4: 4.79 ± 1.85 mg/g, *p* < 0.05). No differences were found in the LPL activity among Myc-MARK4, sh-MARK4 and Vector control in the presence or absence of JW74 (Figure 6E). 

**Figure 7:**
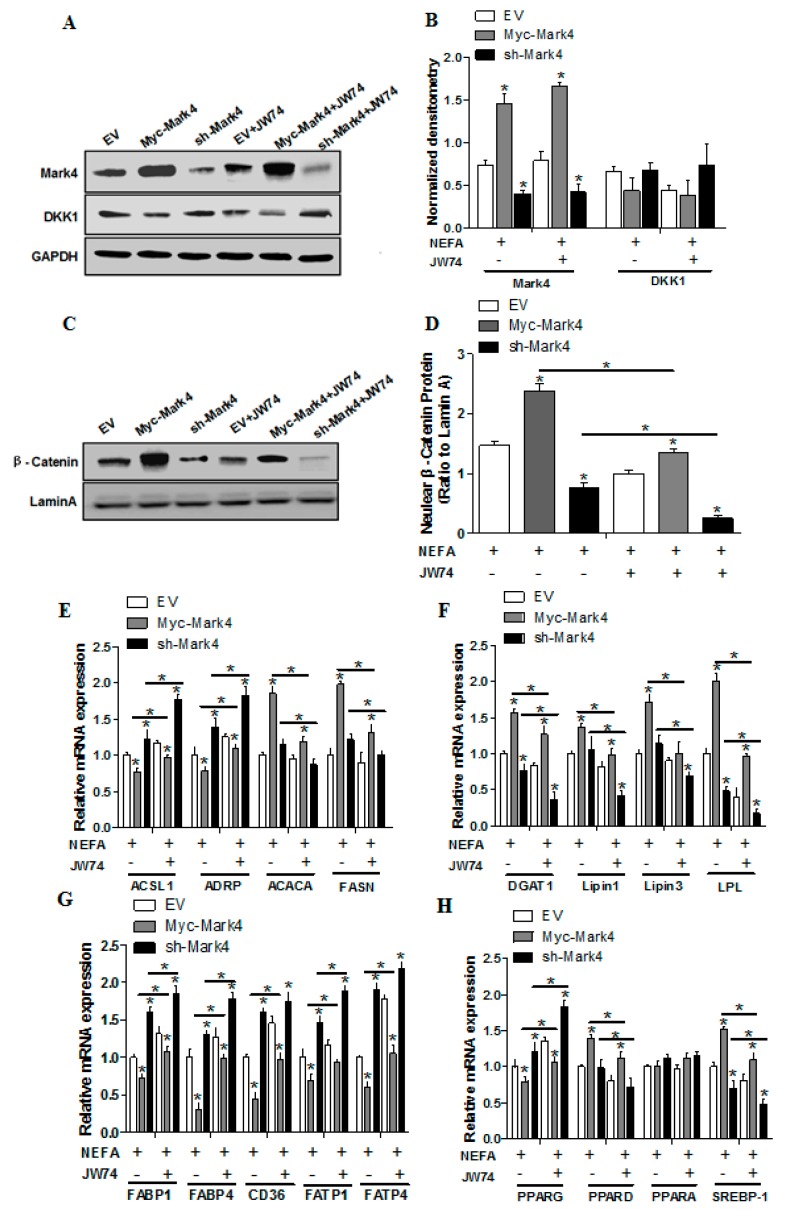
Activation of the WNT/β-catenin pathway by MARK4 promotes lipogenesis in pig primary trophoblast cells challenged with 400 μM NEFA. (**A**–**D**) Representative immunoblots and densitometric quantification for MARK4, DKK1 and β-catenin after transfection with Myc-MARK4, sh-MARK4 for 48 h in primary trophoblast cells isolated from pig placentas. Cells were then incubated with 400 μM NEFA or 10 μM JW74 for 24 h (*n* = 3). (**E**–**H**) Relative mRNA expression of lipid metabolism-related genes (**E** and **F**), fatty acid (FA) transporters (**G**) and regulators of lipid metabolism (**H**) after transfection with Myc-MARK4, sh-MARK4 for 48 h in primary trophoblast cells. Cells were then treated with 400 μM NEFA or 10 μM JW74 for 24 h (*n* = 3). Values are expressed as mean ± SEM.* *p* < 0.05 compared with the control group. Myc-MARK4 group: over expression of MARK4 group, sh-MARK4 group: knock down of MARK4 group, Control: empty vector (EV) group.

We further confirmed the role of WNT signaling on MARK4 activated lipogenesis in trophoblasts by Western blot analysis. Specifically, overexpression of MARK4 increased the protein contents of Mark4 and β-catenin (*p* < 0.05; Figure 7A,C), while no changes were noted for DKK1 expression in Myc-MARK4 or sh-MARK4 treatment in the presence or absence of JW74 (Figure 7B). Despite with JW74 treatment, MARK4 still increased the content of β-catenin within the nucleus (*p* < 0.05; Figure 7D). In accordance with increased receptor-mediated fatty acid accumulation in Myc-MARK4 + JW74 treatment, the mRNA expression of genes associated with fatty acid transport, including ACSL1, ADRP, PPARγ, FATP1, FATP4, CD36, FABP1 and FABP4, were up-regulated in Myc- MARK4 group following exposure to JW74(*p* < 0.05; Figure 7E,G), whereas the mRNA content of genes associated with TG and lipid droplet synthesis, including ACACA, FASN, DGAT1, LPIN1, LPIN3, LPL, PPARδ and SREBP-1c, were decreased in sh-MARK4 group following JW74 treatment (*p* < 0.05; Figure 7E,F,H), in agreement with reduced non- receptor-mediated fatty acid accumulation in sh-MARK4 + JW74 group following exposure to FA + phloretin. 

## 3. Discussion

At present, the MARK4 gene has been widely explored in mammal species [13]. However, such information is still quite limited in *Sus scrofa* (Pig). In this study, the full-length cDNA of MARK4 was characterized from a lean breed swine (Landrace), including an ORF of 2259 bp nucleotides in length, encoding 752 amino acids (AA) residues, in agreement with the previous study [20]. Sequence alignments and phylogenetic analysis showed that MARK4 is highly conserved between *Sus scrofa* (Pig) and other mammals. In addition, several functional sites were also observed, including a protein kinase ATP-binding region, a serine/threonine protein kinase active-site and a protein kinase domain, which represent the typical characters of the protein kinase superfamily [23]. Meanwhile, the catalytic kinase domain (KD), the ubiquitin-associated domain (UBA), the kinase associated domain1 (KA1) and three conserved functional sites (lysine 88 ATP binding site, aspartic 181 active site and threonine 214 phosphorylation site) were also identified through the multiple alignment analysis, which are regarded as the typical structures of microtubule affinity regulatory kinases family [12,13]. Several studies on mammals indicated that the activation of MARK4 is mediated by the major active site (Asp 181) that is activated by phosphorylation of Thr 214 located in the activation loop (T-loop) on protein kinase domain, whereas phosphorylation of Ser 218 in T-loop inactivates MARK4 [24,25]. Accordingly, compared with other mammals, we found the AA sequences of MARK4 protein in *Sus scrofa* has a conserved T-loop sequence, LDTFCGSPP, including the regulatory phosphorylation sites of Thr 214 and Ser 218. Furthermore, the predicted tertiary protein structure of MARK4 in *Sus scrofa* showed high similarity (AA sequence identity is 99%) with that of human (*Homo sapiens*). This was further confirmed by the observation that the key structural residues (Lys 88, Asp 181 and Thr 214) of human MARK4 protein are all well conserved in that of porcine (See Figure 1).

MARK4, the fourth member of microtubule affinity regulatory kinases (MARKs) family, is implicated in the regulation of dynamic biological functions, including glucose homeostasis and energy metabolism [16]. Recently, MARK4 has been found to promote adipogenesis and trigger apoptosis in 3T3-L1 adipocytes [17], suggesting MARK4 may play an important role in regulating lipid metabolism in adipose tissue. In addition, hyperlipidemia associated with obesity has been suggested to contribute to the ectopic lipid accumulation (lipotoxicity) often seen in highly metabolic tissues, including liver, skeletal muscle and placenta [10,11,26], a process that has been implicated as an important mediator of cellular stress and altered tissue function. Regarding the impact of MARK4 on adipogenesis, we hypothesized that Mark4 may potentially stimulate lipid accumulation in other cell types besides placental trophoblast cells from porcine, and this study was designed to investigate the role of MARK4 in modulating lipid metabolic signaling in pig placental trophoblasts in vitro. We found that in pig trophoblast cells MARK4 significantly increased the expression of lipogenic genes, including FASN, ACACA, DGAT1, LPIN1, LPIN3, LPL and SREBP-1c, suggesting increased TG and lipid droplet synthesis by MARK4 expression, as evidenced by dramatically increased lipid droplet accumulation in trophoblast cells. Thus, our data indicated that Mark4 is involved in regulating lipogenesis of pig placental trophoblasts upon the status of lipotoxic insult.

Studies have suggested that activation of PPARγ stimulates fatty acid uptake and fatty acid accumulation in cultured human trophoblast cells [21,27]. However, our data suggests that the stimulating effect of Mark4 on lipid accumulation of trophoblasts is not mediated by increased activation of PPARγ. Furthermore, the MARK4 effect on fatty acid accumulation is unlikely to be due to an activation of LPL activity since MARK4 did not regulate trophoblast LPL activity in vitro. PPARγ is known to be required for placental development and placental uptake of fatty acids [21,28]. Activation of PPARγ regulates gene expression of several proteins involved in lipid transport, including FA transport proteins (FATPs/SLC27As), intracellular FA binding proteins (FABPs), FA translocase (FAT/CD36), adipose differentiation-related protein (ADRP) and Acyl CoA synthase (ACS) [21,27,29]. Our finding showed that MARK4 inhibited the mRNA expression of PPARγ, ADRP, ACSL1, FATP1, FATP4, CD36, FABP1 and FABP4 in cultured trophoblast cells, suggesting impaired FA uptake by trophoblasts in vitro, as evidenced by significantly decreased receptor (transport proteins)-mediated fatty acid accumulation by MARK4. Recent studies determined that PPARγ and MARK4 play an opposing role in adipose inflammation response and oxidative stress [18]. Consistently, we preliminarily determined that activation of PPARγ by PPARγ-specific agonist GW1929 prevented MARK4 from stimulating lipogenesis and non-receptor-mediated lipid accumulation in cultured pig trophoblasts, suggesting that MARK4 promotes lipid synthesis in pig trophoblast cells by inhibiting the PPARγ pathways. However, the precise mechanism for inhibition of PPARγ by MARK4 in regulating lipogenesis of trophoblasts needs to be further studied.

In this study we determined fatty acid accumulation in trophoblast cells which is dependent upon uptake as well as cellular metabolism. As previously documented, WNT signaling pathway is involved in increased placental lipid accretion in obesity-prone rats or obese women [5,10]. In support of the role of WNT signaling in regulating lipid synthesis, our data showed that inhibition of WNT signaling by DKK1 remarkably reduced the mRNA expression of genes associated with TG and lipid droplet synthesis in pig trophoblasts, including DGAT1, LPL, LPIN3 and PPARδ, which is confirmed by decreased lipid droplet accumulation by DKK1. On the contrary, we found that activation of WNT signaling by GSK3β inhibitor LiCL significantly decreased the expression of PPARγ and several FA transporters, including FATP1, CD36, FABP4 and FATP4, in cultured trophoblast cells. Previous studies have shown that β-catenin (a key target of WNT signaling) and PPARγ functionally interact to negatively regulate each other’s activity, and activation of WNT signaling prevents induction of C/EBPα and PPARγ during preadipocyte differentiation [30,31]. Hence, accumulation of fatty acids in pig trophoblast cells in response to lipotoxic insult may be attributed to altered intracellular metabolism of fatty acids rather than changes in cellular uptake.

This study pointed out a significant correlation between MARK4 and WNT signaling. The WNT signal is a cytosolic sensor which activates and promotes β-catenin nuclear translocation and DNA binding [22]. Sun et al. reported that Par-1, the mammalian ortholog of MARKs, is a positive regulator of Wnt/β-catenin pathway in mammalian cells and Drosophila embryos [32]. Consistently, we demonstrated that MARK4 was potent to activate WNT signaling through promoting translocation of β-catenin into the nucleus in cultured pig trophoblasts. It is noticed that activation of WNT signaling pathway by LiCL prevented DKK1 from inhibiting phosphorylation of endogenous Mark4 (Thr214) in trophoblast cells, in agreement with previous studies that WNT signaling stimulates endogenous Par-1 kinase activity [32]. Activation of WNT pathway leads to the phosphorylation of Dishevelled (Dvl) protein, which then inhibits the activity of GSK3β [33]. GSK3β has been shown to inhibit MARK4 protein by phosphorylating the serine residue (Ser218), near the threonine activation site (Thr214) in the activation loop of MARK4 [24]. Therefore, inhibition of GSK3β could be a possible mechanism involved in the activation of MARK4 by WNT signaling. In addition, our experiments employing the WNT specific inhibitor JW74 further confirmed that the WNT pathway is involved in the promotion of lipogenesis via MARK4, suggesting WNT signal is central to MARK4 performing lipid synthesis function in pig trophoblast cells in response to lipotoxic insult.

## 4. Materials and Methods

### 4.1. Experimental Animals and Reagents

For the analysis of full-length cDNA cloning of MARK4 gene and isolation of porcine placental trophoblast cells, samples of placenta from *Sus scrofa* (Landrace) were collected at Research Farm of Nan Jing Agricultural University. The collection of porcine full-term placental tissue was specifically approved by the Laboratory Animal Care and Use Committee of Nan Jing Agricultural University. (SYXK2015-0072, 6 September 2015)

For the isolation of porcine placental trophobalst cells, the following reagents were purchased, including Ham’s F12/Dulbecco’s Modified Eagle Medium(DMEM/F12) (HyClone, Logan, UT, USA), fetal bovine serum (FBS) (HyClone, Logan, UT, USA), Trypsin (Gibco, Grand Island, NY, USA), Phosphate-buffered saline (PBS) (Life Technologies, Grand Island, NY, USA), Bovine serum albumin (BSA) (Amresco, Solon, OH, USA); Percoll (Pharmacia, London, UK), 100× Penicillin-Streptomycin (10,000 U/mL) (Invitrogen, Carlsbad, CA, USA), 100× Insulin–Transferrin–Selenium (ITS; Sigma, Saint Louis, MO, USA) and epidermal growth factor (EGF; Invitrogen, Carlsbad, CA, USA).

### 4.2. Full-length cDNA Cloning of the MARK4 Gene

Total RNA was isolated from the placenta of Landrace sows using RNAiso Plus (TaKaRa, Tokyo, Japan) and then was treated with DNase I using Recombinant RNase-free DNase I kit (TaKaRa, Tokyo, Japan) to degrade genomic DNA. 1% agarose gels electrophoresis and spectrophotometric analysis (260/280 ratio) were used to assess the quantity and quality of isolated RNA.

The cDNA was synthesized with PrimeScript 1 st strand cDNA Synthesis kit (TaKaRa, Tokyo, Japan) using total RNA (1 μg) from the placenta as template and Oligo dT18 as primer according to the manufacturer’s instructions. Degenerated primer pairs of MARK4F/MARK4R (Table 1) were designed based on highly conserved regions from the available sequences of various vertebrate species. PCR amplification was performed with 1 μL of reverse-transcribed (RT) reactions in a total volume of 50 μL and 1 μL Tks Gflex DNA Polymerase (1.25 U/μL; TaKaRa, Tokyo, Japan). The PCR cycling conditions were one cycle of 94 °C for 1 min, 35 cycles of 98 °C for 10sec, 55 °C for 15sec, and 68 °C for 1 min, followed by one cycle of 72 °C for 5 min. The PCR products were purified with MiniBEST Agarose Gel DNA Extraction Kit Ver.4.0 (TaKaRa, Tokyo, Japan) and sequenced by Takara Biotechnology (Dalian) Co.Ltd (Dalian, China). Sequencing was performed in both forward and reverse directions by using an ABI PRISMTM3730XL DNA Sequencer (Applied Biosystems, Waltham, MA, USA). The forward and reverse sequences were assembled using SeqMan NGen15 software in DNASTAR Lasergene 15.2 (DNASTAR, Madison, WI, USA), through which the core fragment of MARK4 gene was obtained. According to the sequence information of this fragment, gene-specific primers were designed for the 3′RACE and 5′RACE.

Rapid amplification of the 3′ end was performed using the 3′-full RACE Core Set with PrimeScriptTM RTase (TaKaRa, Tokyo, Japan) following the manufacturer’s instructions. The primers used for 3′ RACE are shown in Table 1. Firstly, total RNA (1 μg) from placenta was reverse-transcribed using 3′RACE Adaptor (Table 1) as the primer for the synthesis of first strand cDNA. Then, the cDNA was amplified by a specific forward primer MARK4,3-F1 and 3′ RACE Outer Primer containing the anchor sequence. After the first PCR, 1 μL of Outer PCR reactions was re-amplified using 3′ RACE Inner Primer and a specific forward primer MARK4,3-F2. The nested PCR product was separated by electrophoresis using 1% agarose gels and sequenced by the methods aforementioned.

Rapid amplification of the 5′ end was conducted with SMARTerTM RACE 5′/3′ cDNA Amplification Kit (TaKaRa, Tokyo, Japan) according to the manufacturer’s instructions. Briefly, total RNA (1 μg) was reverse-transcribed with a specific reverse primer MARK4,5-R1(Table 1). After the synthesis of first strand cDNA, 2 μL of RT reactions was amplified by prime pairs MARK4,5-R2 and UPM Primer. Then, 1 μL of the first PCR product was used as a template for the nested PCR, which was performed with UPS Primer and MARK4,5-R3. The nested PCR product was separated by 1% agarose gel test and sequenced following the methods aforementioned.

### 4.3. Bioinformatics Analysis

The full-length cDNA of MARK4 gene was obtained using SeqMan NGen15 software in DNASTAR Lasergene (version 15.2) to assemble the core fragment, 3′ end and 5′ end sequences. The resulting nucleotide sequence was edited and analyzed by Open Reading Frame (ORF) Finder on NCBI (https://www.ncb-i.nlm.nih.gov/orffinder), and then translated into amino acids (AA) using standard genetic codes. The molecular weight (MW) and isolectric point (PI) of the Mark4 protein were predicted using the compute PI/MW software at https://web.expasy.org/compute_pi. Multiple alignments were generated by the MegAlign 15 program in DNASTAR Lasergene (version 15.2). The secondary and three-dimensional (3D) structures of Mark4 protein were predicted by the SABLE program (http://sable.cchmc.org) and the SWISS-MODEL program (https://swissmodel.expasy.org) as previously described [34], respectively. Illustration of the MARK4 model was performed in PyMOL 2.2 program (https://pymol.org). Phylogeny tree was inferred by the MEGA7 program, and distance analysis was conducted using the Neighbour-Joining (NJ) algorithm. 1000 bootstrap-replications were generated to evaluate the reliability for each code.

### 4.4. Porcine Placental Trophobalst Cell Isolation and Culture

The isolation and culture of porcine placental trophobalst cells were performed as previously described with some modifications [35]. Briefly, placental villous tissue, obtained from vaginal delivery, were dissected from fetal amnion and rinsed thoroughly in cold PBS containing 100 U/mL penicillin and 100 μg/mL streptomycin, and then cut into 1–3 mm 3 pieces. The tissue fragments were digested with 0.125% (*w/v*) Type I collagenase (trypsin) at 37 °C for 30 min with continuous shaking, followed by filtration through a 70 μm cell strainer. The filtrate was further purified by Percoll gradient centrifugation. Placental trophoblast cells were collected from the appropriate layers between 35% and 45% Percoll density gradient separated layers, and cultured in DMEM/F12 supplemented with 10% FBS, 1% (*v/v*) ITS, 10 ng/mL of EGF, 100 U/mL penicillin and 100 μg/mL streptomycin at 37 °C under 5% CO2 as previously described [35]. The purity of trophoblast cells isolated from full-term placentas was determined by flow cytometry as previously described [36], using FITC fluorescein-labeled antibody against cytokeratin-7 (Santa Cruz Tech, Dallas, CA, USA) as a specific marker of trophoblast cells.

### 4.5. Cell Transfection and Drug Treatment

DNA constructs including Myc-MARK4 and Flag-DKK1 were made by Generay Biotech Company (Shanghai, China) using pEGFP-N1 expression vector. shRNA sequences against MARK4 or DKK1 were contrived and synthesized by Genepharma Company (Shanghai, China) using pGpU6/GFP/Neo shRNA expression vector. After transfection efficiency detection, the optimal shRNA of MARK4 or DKK1 was chosen and named sh-MARK4 and sh-DKK1. Cells were plated at a concentration of 6 × 10^5^–2 × 10^6^/dish in 60-mm dishes. 2 μg interference or expression plasmids DNA were mixed with X-treme GENE HP Reagent (Roche, Basel, Switzerland) and Opti-MEMI media (Invitrogen, Carlsbad, CA, USA) following the instruction. The transfection mixture was then added into each dish for 48 h to allow the expression of DNA or shRNA constructs described above.

In order to induce lipid accumulation in trophoblast cells in vitro, cells were treated with 400 μM Fatty Acid (FA) Supplement containing 2 mol of linoleic acid and 2 mol of oleic acid per mole of albumin (L9655; Sigma-Aldrich, Saint Louis, MO, USA) in triplicate as previously described [10,37]. The optimal treatment concentration of 400 μM was chosen based on results of concentration gradient studies (Figure S5) indicating that fat accumulation was significantly increased by 50, 100 and 200 μM fatty acids when compared to 0μM, with the most significant increase following the 400 μM treatment. Treatment media without fatty acids was added with bovine serum albumin (FA free) to maintain the same osmolarity. In some experiment, cells were treated with one of the following specific agonists or inhibitors: 2 μM GW1929 (PPARγ-specific agonist; MCE, Shanghai, China), 20 μM LiCL (GSK3β inhibitor; Millipore, Billerica, MA, USA), or 10 μM JW74 (WNT signaling pathway specific inhibitor; MCE, Shanghai, China) for the amount of time specified in the individual figures.

### 4.6. Oil Red O Staining

After 24 h of FA treatment, cells were fixed in 4% paraformaldehyde for 30 min at room temperature for Oil Red O staining. Each well was then briefly washed in PBS and 60% isopropanol and then stained for 10 min in a 60% working Oil Red O solution (Sigma-Aldrich). For quantification of Oil Red O staining, cells were extracted by 100% isopropanol for colorimetric analysis at an optical density of 490 nm as previously described [10].

### 4.7. Cell Viability and Reactive Oxygen Species (ROS) Assay

Cell viability was detected using cell counting kit-8 (CKK-8; KeyGen BioTECH, Nanjing, China). The isolated cells were seeded into 96-well plates at a density of 5 × 10^3^ and cultured with 0, 400 and 500 μM fatty acids for the amount of time specified in Figure S5, respectively. 10 μL CKK-8 solution was then added into each well and incubated for 2 h at 37 °C. Absorbance was measured at 450 nm using a Multiskan Go Microplate Spectrophotometer (Thermo Scientific, Waltham, MA, USA).

The intracellular level of ROS test was performed using Oxygen Species Assay Kit (KeyGen BioTECH, Nanjing, China) according to the manufacturer’s instructions. Briefly, the dye loading was performed by incubating the cells with 10 μM 2′, 7′-dichlorofluorescin diacetate (DCFH-DA) at 37 °C for 1 h. The production of ROS was examined using a Luminescence Spectrophotometer (Promega Corporation, Madison, WI, USA) by measuring the fluorescence intensity of DCF at emission wavelength of 525 nm.

### 4.8. Lipid Accumulation Assay

The Bodipy 493/503 lipid probes (D-3922; Thermo Scientific) was used to visualize fatty acid accumulation in cultured trophoblast cells as previously described [38]. Briefly, cells were washed in PBS and 4% paraformaldehyde in PBS was added to fix the cells for 30 min at room temperature. After fixation, cells were washed in PBS containing 0.1% Triton X-100 for 5 min. Bodipy dye was diluted in PBS at a concentration of 10 μg/mL and applied to cells for 15 min. For nuclei staining, 10 μg/mL of 4′, 6-diamidino-2-phenylindole (DAPI) solution was incubated with each sample for 30 min, and then the samples were examined on confocal laser scanning microscope (Zeiss LSM 700 META, Jena, Germany). For quantification of lipid accumulation, triglyceride (TG) content in cultured trophoblast cells was evaluated with a spectrophotometer (Thermo Scientific) at 510 nm using Tissue Triglyceride Assay Kit (APPLYGEN, Beijing, China) as previously described [17]. Because phloretin blocks receptor (transport proteins)-mediated fatty acid transport and accumulation [39,40] it was used to determine receptor-mediated fatty acid accumulation by subtracting the TG content in the presence of phloretin (500 μM) from those in the absence of phloretin as previously described [21].

### 4.9. Immunofluorescence Assay

β-catenin immunofluorescence analysis was performed as previously described [10]. Briefly, cells were grown on coverslips, fixed with 4% paraformaldehyde for 30 min and permeabilized with 0.25% Triton X-100 for 10 min. After blocked with 5% BSA-supplemented PBS for 1 h, cells were incubated overnight at 4 °C with rabbit anti-β-Catenin primary antibody (8480, dilution 1:300, Cell Signaling Technology, Danvers, MA, USA), followed by incubation of Goat anti-rabbit Cy3 fluorescein-labeled secondary antibody (BA1032, dilution 1:500, Boster, China). Meanwhile, the cell nuclei were counterstained with 4′, 6-diamidino-2-phenylindole for 10 min, and then the samples were mounted on glass slides and examined on confocal laser scanning microscope (Zeiss LSM 700 META, Jena, Germany). Quantification of the fluorescence intensity from the red channel (β-Catenin) was performed using the Image J software (NIH Image).

### 4.10. Measurement of LPL Activity

For LPL activity detection, cells were harvested after the medium removed, washed with ice-cold PBS and lysed with cell lysis buffer (20 mM Tris, 150mM NaCl, 1% Triton X-100). The lysate was centrifuged at 10,000× *g* for 5 min at 4 °C. Then LPL enzyme activity was measured in the supernatant by the enzyme fluorescence method using Biovision LPL Activity assay kit (Biovision Incorporated, Milpitas, CA, USA) according to the manufacturer’s instructions as previously described [9]. Results were normalized to the amount of protein (mU per mg of bulk cellular protein). Protein concentration was determined using Pierce BCA Protein Assay Kit (Thermo Scientific, Waltham, MA, USA) according to the manufacturer’s instructions.

### 4.11. Real-time Quantitative PCR Analysis

Total RNA was extracted from cultured cells with the High Pure RNA tissue kit (Omega Bio-Tek, Norcross, GA, USA) and 500 ng of total RNA was reverse transcribed using PrimeScript RT Master Mix Kit (TaKaRa, Tokyo, Japan). Real-time RT-PCR was conducted on the Step One Plus Real-Time PCR System (ABI, Waltham, MA, USA) with the following program: 95 °C for 30 sec, 95 °C for 5 sec, 60 °C for 30 sec, 95 °C for 15 sec, 60 °C for 1 min, and 95 °C for 15 sec, with 40 cycles of steps 2 and 3. Primers were synthesized by Invitrogen (Shanghai, China). Amplication was performed in 25µl reaction system containing specific primers (Table S1) and SYBR Premix Ex Taq II (TaKaRa, Tokyo, Japan). Relative gene expression was calculated using the comparative Ct method with the formula 2^−ΔΔCt^ [41]. The two reference genes GAPDH and HPRT1 were used. The geometric mean of relative gene expression was calculated and used for further analysis as previously reported [42].

### 4.12. Protein Extraction and Western Blotting Analysis

Total protein from cultured trophobalst cells was extracted using cell lysis buffer (Beyotime Co, China) by procedures as previously described [38]. Nuclear protein isolation was performed using Nuclear and Cytoplasmic Protein Extraction Kit (KenGEN BioTECH, Nanjing, China) according to the manufacturer’s instructions as previously reported [11]. The concentration of protein was quantified using BCA Protein Assay kit (Thermo Scientific, Waltham, MA, USA). Proteins (50 μg) were separated by SDS-PAGE and transferred to PVDF nitrocellulose membrane (Bio-Rad Laboratories, Hercules, CA, USA). After blocking in 5% fat-free milk for 1 h at room temperature, the membranes were incubated with rabbit anti-Mark4(4834, 1:1000 dilution, Cell Signaling Technology, Danvers, MA, USA), β-Catenin (8480, dilution 1:1000, Cell Signaling Technology), GAPDH (2118, dilution 1:1000, Cell Signaling Technology), and Phospho-Mark4 (SAB4504258, 1:500 dilution, Sigma, Saint Louis, MO, USA) antibody, Goat anti-DKK1 (LS-B194, dilution 1:1000, LifeSpan BioSciences, Seattle, WA, USA) antibody, or Mouse anti-LaminA (sc-376248, dilution 1:1000, Santa Cruz Biotechnology, Dallas, TX, USA) antibody overnight at 4 °C, followed by incubation with Donkey anti-goat, Goat anti- mouse or rabbit IgG horseradish peroxidase (HRP)-conjugated secondary antibodies (HAF109,HAF007 and HAF008, dilution 1:2000, RD SYSTEMS, Minneapolis, USA) for 1 h at room temperature. Proteins were visualized using the LumiGLO Reagent and Peroxide system (Cell Signaling Technology, Danvers, USA), and then the blots were quantified using Bio-Rad ChemiDoc imaging system (Bio-Rad Laboratories, Hercules, USA). Band density was normalized according to the GAPDH content.

### 4.13. Statistical Analysis

All the data were obtained from at least three independent experiments. Statistical analyses were conducted using SPSS Statistics 20.0 software (IBM SPSS, Armonk, NY, USA). Data were analyzed using One-way ANOVA for comparisons among groups, followed by Duncan test. Results were expressed as means ± SEM. A *p*-value < 0.05 was considered statistically significant, and very significant was indicated when *p* < 0.01.

## 5. Conclusions

**Figure 8:**
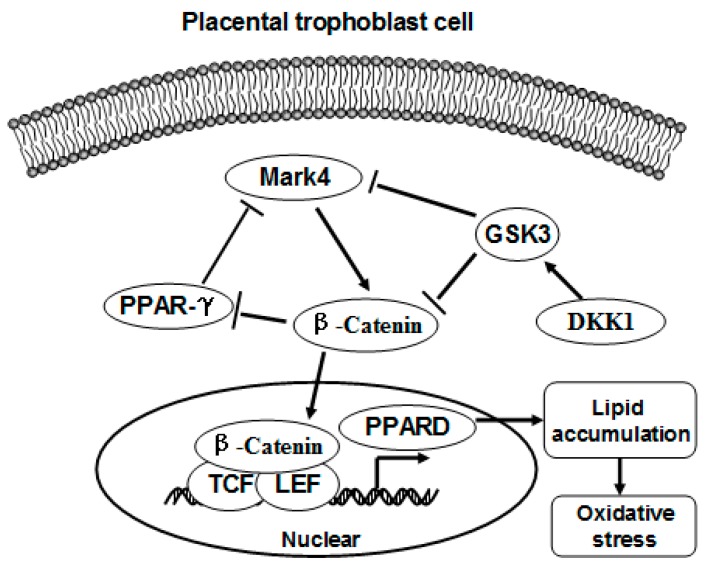
A proposed model for role of MARK4 in regulating lipogenesis in pig placental trophoblast cells. MARK4 promotes lipogenesis by activating WNT/β-catenin signaling pathway. Arrows indicates a positive regulation and bar-headed lines show negative regulation. Interactions depicted are based on studies performed in various tissues (in some cases placenta) and have been previously published.

In summary, our present study demonstrates that MARK4 stimulates fatty acid accumulation in porcine trophoblast cells, which could contribute to a lipotoxic placental milieu in conditions associated with elevated maternal fatty acids such as excessive back-fat during pregnancy of sows. Moreover, WNT/β-catenin signal is essential for MARK4 promoting lipogenesis in pig placental trophoblasts (Figure 8). Thus, our results indicate that MARK4 has potential as a regulator of lipotoxicity associated with maternal obesity in the pig placenta.

## Figures and Tables

**Table 1:** Primers for the cDNA cloning of MARK4 gene.

Primers	Sequence, 5′-/-3′	Use
MARK4-F	CAACGATCGGAACTCGGACA	Used with MARK4-R for RT-PCR of core fragment
MARK4-R	ATTTGGCAACAGGGACGGGC	Used with MARK4-F
3′RACE Adaptor	CTGATCTAGAGGTACCGGATCC(T)_16_	Used for synthesis of the first- strand cDNA for 3′RACE
MARK4,3-F1	CAAGCGCAGCCCAACCAGCACAG	Used with 3′Outer Primer for first PCR of 3′RACE
3′Outer Primer	TACCGTCGTTCCACTAGTGATTT	Used with MARK4,3-F1
MARK4,3-F2	ACAAGGCAGAGATCCCAGAGCGA	Used with 3′Inner Primer for nested PCR of 3′RACE
3′Inner Primer	CGCGGATCCTCCACTAGTGATTTCA-CTATAGG	Used with MARK4,3-F2
MARK4,5-R1	AGCTTCACAATGTTGGGGTGGTT	Used for synthesis of the first-strand cDNA for 5′RACE
MARK4,5-R2	TGGGGTTCAGCTGGGTTTTGTCG	Used with UPM Primer for first PCR of 5′RACE
UPM Primer	CTAATACGACTCACTATAGGGCAA-GCAGTGGTATCAACGCAGAGT	Used with MARK4,5-R2
MARK4,5-R3	AGGATGTGCCGGGCCAGCTTGAC	Used with UPS Primer for nested PCR of 5′RACE
UPS Primer	CTAATACGACTCACTATAGGGC	Used with MARK4,5-R3

## Supplementary Materials

Supplementary Materials can be found at https://www.mdpi.com/1422-0067/20/5/1206/s1, Figure S1: The full-length cDNA of MARK4 gene in porcine and the deduced amino acid sequence, Figure S2: Clustal W alignment of the MARK4 protein from pig and other organisms, Figure S3: The secondary structure of the MARK4 protein in porcine constructed by the SABLE program, Figure S4: Phylogenetic tree based on MARK4 sequences made by MEGA 7.0 software using the neighbor-joining method, Figure S5: Identification of optimal fatty acid (FA) concentration for induction of FA accumulation in pig primary cytotrophoblasts, Table S1: Primer sets used for real-time PCR.

## Abbreviations

ACACA	Acetyl-CoA carboxylase
ADRP	Adipose differentiation-related protein
ACSL1	Acyl CoA synthase long chain family member 1
cDNA	Complementary DNA
CD36	Fatty acid translocase
DGAT1	Diglyceride acyltransferase 1
DKK1	Dickkopf family protein1
FASN	Fatty acid synthase
FA	Fatty acid
FATP1	Fatty acid transport protein 1
FATP4	Fatty acid transport protein 4
FABP1	Intracellular fatty acid binding protein 1
FABP4	Intracellular fatty acid binding protein 4
FITC	Fluorescein isothiocyanate
GSK3β	Glycogen synthase kinase 3 beta
GAPDH	Glyceraldehyde-3-phosphate dehydrogenase
HPRT1	Hypoxanthine phosphoribosyltransferase 1
LPL	Lipoprotein lipase
LPIN1	Phosphatidic acid phosphatase 1
LPIN3	Phosphatidic acid phosphatase 3
MARK1	Microtubule affinity-regulating kinase 1
MARK2	Microtubule affinity-regulating kinase 2
MARK3	Microtubule affinity-regulating kinase 3
MARK4	Microtubule affinity-regulating kinase 4
ORF	Open reading frame
PPARγ	Peroxisome proliferators-activated receptor gamma
PPARD	Peroxisome proliferators-activated receptor delta
PPARA	Peroxisome proliferators-activated receptor alpha
RACE	Rapid Amplification of cDNA Ends
RT-PCR	Reverse transcription-polymerase chain reaction
SREBP-1c	Sterol regulatory element binding protein 1c
TG	Triglyceride
