# Supplementary material for: Molecular Characterization of Microtubule Affinity-Regulating Kinase4 from Sus scrofa and Promotion of Lipogenesis in Primary Porcine Placental Trophoblasts
Source: Int J Mol Sci. 2019 Mar 9;20(5):1206. doi: 10.3390/ijms20051206 (PMC6429113; doi:10.3390/ijms20051206)
Supplement: Supplementary file 1 [file ijms-20-01206-s001.zip › ijms-447478-SI/Supplementary Materials-R/Figure S1.doc]

1	GCCGCCGCCCGGGCCGGCTCCGCGCCCCCTCCCTGGCCCCCGCCCGCCGGCCTGCCCGCC

61	GCCCCCATGGCGCCCCCAGCCCGAGCTGTGCGGGGCCACTAGGACCCTAGGCACCCCCTT

121	CCCTCCCCCGCCCTGCCCCCTCTCCCACGGCGCGAACCCGGGTGTCCACGGCGCCCAGCT

181	TTTGAGCTCGCGTCTCCAGGCCGGTGGGGGGGGAGGGGCAGAGAGGGGACCCCCCCCGGA

241	CCCCCGCCCCCCCCCCGACCTGGCCGCCCAGTCCCCTGGGACCCGGAGAAAATGTCTTCG

1	M	S	S	301	CGGACGGCGCTGGCCCCGGGCAACGATCGGAACTCGGACACGCATGGCACCCTGGGCAGT

4	R T	A	L	A	P	G	N	D	R N	S	D	T	H	G T	L G	S

361	GGCCGCTCCTCTGACAAGGGACCGTCCTGGTCCAGCCGCTCCCTGGGTGCCCGCTGCCGG

24	G	R S	S	D	K	G	P	S	W	S	S	R	S	L	G	A R C	R

421	AACTCCATCGCCTCCTGTCCTGAGGAGCAGCCACACGTGGGCAACTACCGCCTGCTGAGG

44	N	S	I	A	S	C	P	E	E	Q	P	H V	G	N Y	R	L	L	R

481	ACCATTGGGAAGGGCAACTTTGCCAAAGTCAAGCTGGCCCGGCACATCCTTACCGGCCGG

64	T	I	G	K	G	N F	A	K	V	K	L A	R	H	I	L	T	G	R

541	GAGGTCGCCATCAAGATTATCGACAAAACCCAGCTGAACCCCAGCAGCCTGCAGAAACTG

84	E	V	A	I	K	I I	D	K	T	Q	L N	P	S	S	L	Q	K	L

601	TTCCGAGAAGTCCGCATCATGAAGGGCCTGAACCACCCCAACATTGTGAAGCTCTTCGAG

104	F	R	E	V	R	I M	K	G	L	N	H P	N	I	V K	L	F	E

661	GTCATCGAGACAGAGAAGACGCTGTACCTGGTGATGGAATACGCAAGTGCTGGAGAAGTG

124	V	I	E	T	E	K T	L	Y	L	V	M E	Y A	S	A	G	E V

721	TTTGACTACCTCGTGTCGCACGGCCGCATGAAGGAGAAGGAAGCTCGAGCCAAGTTCCGA

144	F	D	Y	L	V S H	G	R M	K	E	K	E	A	R	A	K	F R

781	CAGATTGTGTCGGCTGTGCACTACTGTCACCAGAAAAACATTGTCCACAGGGACCTGAAG

164	Q	I	V	S	A V H	Y C	H	Q	K	N I	V	H	R D	L K

△

841	GCGGAGAACCTCTTGCTGGACGCCGAGGCCAACATCAAGATCGCCGACTTTGGCTTCAGC

184	A	E	N	L	L L D	A E	A	N	I	K I	A	D	F	G	F S

901	AATGAGTTCACACTGGGCTCGAAGCTGGACACATTCTGCGGGAGCCCCCCGTACGCCGCC

204	N	E	F	T	L G S	K	L	D	T	F	C G	S	P	P	Y	A A

961	CCGGAGCTGTTCCAGGGCAAGAAGTACGACGGGCCGGAGGTGGACATCTGGAGCCTGGGC

224	P	E	L	F	Q G K	K	Y	D	G	P	E V	D	I	W S	L G

1021 GTTATCCTGTACACCCTCGTCAGCGGCTCCCTGCCCTTCGACGGGCACAACCTCAAGGAG

244	V	I	L	Y	T	L	V S	G	S	L	P F	D	G H	N	L K	E

1081 CTGCGGGAGCGAGTCCTCAGAGGGAAGTACCGGGTCCCTTTCTACATGTCAACAGACTGT

264	L	R	E	R	V	L	R	G	K Y	R V	P	F	Y	M	S T	D C

1141 GAGAGCATCCTGCGGAGATTTTTGGTGCTGAATCCAGCTAAACGCTGTACTCTGGAGCAA

284	E	S	I	L	R	R	F	L	V	L	N P	A	K	R	C	T L	E	Q

1201 ATCATGAAAGACAAGTGGATCAACATCGGCTACGAGGGCGAGGAGCTGAAGCCATACACG

304	I	M	K	D	K	W	I	N	I	G	Y E	G	E	E	L	K	P	Y T

1261 GAGCCTGAGGAGGACTTCGGGGACACCAAACGAATTGAGGTGATGGTGGGTATGGGCTAC

324	E	P	E	E	D	F	G	D T	K	R	I	E	V	M V	G M	G Y

1321 ACGCGGGAAGAAATCAAAGAGGCCTTGACCAGCCAGAAGTACAACGAAGTGACCGCCACC

344	T	R	E	E	I	K	E	A	L	T	S	Q K	Y	N	E	V T	A	T

1381 TACCTCCTGCTGGGCAGGAAGACTGAGGAGGGTGGGGACCGGGGCGCCCCGGGGCTGGCC

364	Y	L	L	L G	R	K	T	E	E	G	G D	R	G	A	P G	L	A

1441 CTGGCACGGGTGCGGGCGCCCAGCGACACCACCAATGGAACAAGCTCCAGCAAAGGCACC

384	L	A	R	V R	A	P	S	D	T	T	N G	T	S	S	S K	G T

1501 AGCCACAGCAAAGGGCAGCGGAGCTCCTCCTCCACCTACCACCGCCAGCGCAGGCACAGC

404	S	H	S	K	G Q	R	S	S	S	S	T	Y	H	R Q	R R	H S

1561 GACTTCTGTGGTCCGTCTCCCGCACCCCTGCACCCCAAGCGCAGCCCAACCAGCACAGGG

424	D	F	C	G	P	S	P	A	P	L	H	P	K	R	S P	T	S	T G

1621 GAGGCGGAACTGAAGGAGGAGCGCCTGCCAGGCCGGAAGGCAAGCTGCAGTGCTGCTGGG

444	E	A	E	L	K	E	E	R	L	P	G	R	K	A	S C	S A	A G

1681 AGCGGGAGCCGAGGGCTGCCCCCCTCCAGCCCCATGGTCAGCAGTGCCCACAACCCCAAC

464	S	G	S	R	G	L	P	P	S	S	P	M	V	S	S A H N	P N

1741 AAGGCAGAGATCCCAGAGCGACGGAAGGACAGCACGAGCACCCCTAACAACCTCCCTCCC

484	K	A	E	I	P	E	R	R K	D	S	T	S	T	P N	N L	P P

1801 AGCATGATGACCCGCAGAAACACCTATGTTTGCACAGAACGCCCAGGGGCTGAGCGCCCG

504	S	M	M	T	R	R	N	T	Y	V C	T	E	R	P G	A	E R P

1861 TCCCTGTTGCCAAATGGCAAAGAAAACAGCTCGGGTACCCCACGGGTGCCCCCCGCCTCC

524	S	L	L	P	N G	K	E	N	S	S	G	T	P	R	V	P	P A S

1921 CCCTCCAGTCACAGCCTGGCTCCCCCATCGGGGGAGCGGAGCCGCCTGGCACGCGGCTCC

544	P	S	S	H	S L	A	P	P	S	G	E	R	S	R	L	A R G S

1981 ACCATCCGCAGCACCTTCCACGGTGGCCAGGTCCGAGACAGGCGGGCAGGGGGCGGGGGA

564	T	I	R	S	T	F	H	G G	Q	V	R	D	R	R A	G G G G

2041 GGTGGGGGCGTGCAGAACGGCCCCCCCGCCTCTCCCACCCTGGCCCACGAGGCCACGCCC

584	G	G	G	V	Q	N	G	P P	A	S	P	T	L A	H	E A T P

2101 CTGCCCACTGGGCGGCCCCGCCCCACCACCAACCTCTTCACCAAGCTGACCTCCAAACTG

604	L	P	T	G	R	P	R	P T	T	N	L	F	T	K	L T	S K L

2161 ACCCGAAGGGTCACAGACGAACCTGAGAGAATCGGGGGACCTGAGGTCACAAGTTGCCAT

624	T	R	R	V	T	D	E	P	E	R	I G	G	P	E	V T	S	C	H

2221 CTACCTTGGGATCAAACGGAAACCGCCCCCCGGCTGCTCCGATTCCCCTGGAGTGTGAAG

644	L	P	W	D	Q	T	E	T	A	P	R L	L	R	F	P W	S	V K

2281 CTGACCAGCTCGCGCCCGCCCGAGGCCCTGATGGCAGCTCTGCGCCAGGCCACCGCGGCC

664	L	T	S	S	R	P	P	E	A	L	M A	A	L	R Q	A	T A A

2341 GCCCGCTGCCGCTGCCGCCAGCCACAGCCGTTCCTGCTGGCCTGCCTGCACGGGGGTGCG

684	A	R	C	R	C	R Q	P	Q	P	F	L	L	A	C L	H	G G A

2401 GGCGGGCCCGAGCCCCTGTCCCACTTCGAAGTGGAGGTCTGCCAGCTGCCCCGGCCGGGC

704	G	G	P	E	P	L S	H	F	E	V	E	V	C	Q	L	P	R P G

2461 CTGCGGGGAGTTCTCTTCCGCCGCGTGGCGGGCACCGCCCTGGCCTTCCGCACCCTCGTC

724	L	R	G	V	L	F R	R V	A	G T	A	L	A	F R	T L V

2521 ACCCGCATCTCCAACGACCTCGAGCTCTGAGCCGCCAGGGCCCTGGGCCCCCTCCTCTTC

744	T	R	I	S	N	D L	E	L *

2581 CTCTTCCTCGCCCCCTTCGCTTCAGCAGGAGGGAAAGGGGGCCAAGGAGGGGGTCTCCCT

2641 TTGGCATCGCCTCCGTTCCCCTGAGACCAGTGCGGGGGCAGAGCCTGTCCCCTCTGCTGT

2701 TCTCCGAGGCCACCGGGCAGGAGAGAAGAGCAAAGGGCCTTCGCGGGGCGGCGGGGAGCT

2761 GGCACCTTCCTAGAGCCTCCAGCCCATCTCGTCCCCCGTCCCCCTGCCGTGGGGCCCTGA

2821 GGAGACTGCAGGGACGGAGCAGAAAGGGAAACTGAGGAAACTCCTCTAACCCCGCCAGCT

2881 CAAGAACTAGGCCTTGAGCAAGGGCAGGAAGCGTTGCTGAGCCTGAAGACTGGAGCACGG

2941 GGGGCTGCTGGGAGCAGGGGCCAGAGAGGCAGGTTCCTCCCCTCCCTCTCCCCTCCTGCT

3001 CGAACCCCCCCTTACTGCCCCAGGCTGGCGCGGGGCACTTTGTACAAATCCTTGTAAATA

3061 CACCCACACCCTCCCCTCTGCAGAGATCTCTTGAGGAGCTGCCGCTCTCACCTCTGGTTT

3121 TTAAGTTATTACACCCCCCTCCTCCTGTCAGCCTCCCTCACCTGCAGCCTGTTGCCCAAT

3181 AAACTTAGGAGAGTCCCCCCTCCCCAAAAAAAAAAA

Figure S1 The full-length cDNA of MARK4 gene in porcine and the deduced amino acid sequence. Red letters indicate the translated region and no colored letters indicate the untranslated region. The protein kinase ATP-binding region signature is underlined. The serine/threonine protein kinase active-site signature is dot underlined. The protein kinase domain profile is shaded. The triangles indicate the proton acceptor. The nucleotide sequence was submitted to NCBI GenBank, accession number: MH926032.
