# Supplementary material for: Molecular Characterization of Microtubule Affinity-Regulating Kinase4 from Sus scrofa and Promotion of Lipogenesis in Primary Porcine Placental Trophoblasts
Source: Int J Mol Sci. 2019 Mar 9;20(5):1206. doi: 10.3390/ijms20051206 (PMC6429113; doi:10.3390/ijms20051206)
Supplement: Supplementary file 1 [file ijms-20-01206-s001.zip › ijms-447478-SI/Supplementary Materials-R/Figure S3.doc]

Figure S3. Clustal W alignmet of the MARK4 protein from pig and other organisms. Identical animal acids are marked in red and homologous amino acids in yellow. Arrow indicates the activation loop (T-loop).The green, blue and pink box indicate catalytic kinase domain (KD), ubiquitin-associated domain (UBA) and kinase associated domain1 (KA1), respectively. The lysine 88 ATP binding site, aspartic 181 active site and threonine 214 phosphorylation site are in black oval.The accession numbers for the sequences are as follows: American beaver(*Castor Canadensis*)(XP020028554.1); Horse (*Equus caballus*)(XP023505667.1); Human(*Homo sapiens*) (NP001186796.1); House mouse (*Mus musculus*)(NP758483.1); Norway rat (*Rattus norvegicus*) (NP001178000.1); Zebra fish (*Danio rerio*)(XP005157657.1); Domestic guinea pig (*Cavia porcellus*) (XP013002354.1); Dingo (*Canislupusdingo*)(XP025280449.1); Pig (*Sus scrofa*) (MH926032); Chimpanzee(*Pan troglodytes*)(XP 016791695.1); Davids myotis (*Myotis davidii*) (XP 015416684.1).
