# Supplementary material for: Molecular Characterization of Microtubule Affinity-Regulating Kinase4 from Sus scrofa and Promotion of Lipogenesis in Primary Porcine Placental Trophoblasts
Source: Int J Mol Sci. 2019 Mar 9;20(5):1206. doi: 10.3390/ijms20051206 (PMC6429113; doi:10.3390/ijms20051206)
Supplement: Supplementary file 1 [file ijms-20-01206-s001.zip › ijms-447478-SI/Supplementary Materials-R/Figure S4.doc]

Fig.S4 Phylogenetic tree based on MARK4 sequences made by MEGA 7.0 software using the neighbor-joining method. The evolutionary distances were computed using the Poisson correction method. The numbers represent bootstrap percentages. The topology was tested using bootstrap analysis (1000 replicates). Genbank Accession numbers of the sequences are indicated in figure.
