# Supplementary material for: Molecular Characterization of Microtubule Affinity-Regulating Kinase4 from Sus scrofa and Promotion of Lipogenesis in Primary Porcine Placental Trophoblasts
Source: Int J Mol Sci. 2019 Mar 9;20(5):1206. doi: 10.3390/ijms20051206 (PMC6429113; doi:10.3390/ijms20051206)
Supplement: Supplementary file 1 [file ijms-20-01206-s001.zip › ijms-447478-SI/Supplementary Materials-R/Figure S5.doc]

Figure S5. Identification of optimal fatty acid (FA) concentration for induction of FA accumulation in pig primary totrophoblast cells. (A) Representative Oil Red O staining of trophoblasts incubated for 24h in the presence of 0, 50, 100, 200, 400, 500 μM of NEFA. (B) Quantification of the Oil Red O staining (n=3). (C) Primary trophoblasts isolated from pig placentas were cultured and incubated for 0 h, 12 h, 24 h, 36 h and 48 h in the presence of 400 nM or 500μM NEFA. Cell viability was detected by MTT (n = 3). (D) The relative ROS activity of the primary trophoblast cells incubated for 24 h in the presence of 400 or 500μM NEFA (n = 3). Values are expressed as mean ± SEM. * *P* ＜0.05 compared with the control group. No significance is shown as ns. Control: no NEFA group. NEFA: Non-esterified fatty acids.
