# Supplementary material for: Molecular Characterization of Microtubule Affinity-Regulating Kinase4 from Sus scrofa and Promotion of Lipogenesis in Primary Porcine Placental Trophoblasts
Source: Int J Mol Sci. 2019 Mar 9;20(5):1206. doi: 10.3390/ijms20051206 (PMC6429113; doi:10.3390/ijms20051206)
Supplement: Supplementary file 1 [file ijms-20-01206-s001.zip › ijms-447478-SI/Supplementary Materials-R/Table S1.doc]

Table S1 Primer sets used for real-time PCR

| Gene name^†^ | Productsize (bp) | Direction | Sequence, 5’-/- 3’ |
| --- | --- | --- | --- |
| ADRP | 135 | Forward: | AGGATCAGTATCCCTACTTGAA |
|  |  | Reverse: | AGTGTTGGCAATGGCAATCT |
| PPARD | 167 | Forward: | AAGGCATCAGGCTTCCACTAC |
|  |  | Reverse: | CATTTCTGGAAGCGGCAGTA |
| FASN | 98 | Forward: | CACTCCAAGCAGGCGAACAC |
|  |  | Reverse: | CGAAGGGAAGCAGGGTTGAT |
| ACACA | 129 | Forward: | ATGTTTCGGCAGTCCCTGAT |
|  |  | Reverse: | TGTGGACCAGCTGACCTTGA |
| DGAT1 | 96 | Forward: | TGGTGGAACTCGGAGTCTGTCA |
|  |  | Reverse: | GCCGGAGCATGGGCTTGTAG |
| LIPIN1 | 149 | Forward: | TCCGACATTGACGGAACCAT |
|  |  | Reverse: | GCACGAGCCGAGCAGTAGAG |
| LIPIN3 | 116 | Forward: | GACAGTGTTTGGGACAGTGAAGG |
|  |  | Reverse: | AGGGTGAGCATCGGAAGGAG |
| PPARG | 184 | Forward: | GATTTCTCCAGCATTTCCA |
|  |  | Reverse: | GCTCTTCGTGAGGTTTGTT |
| SREBP-1c | 218 | Forward: | GCGACGGTGCCTCTGGTAGT |
|  |  | Reverse: | CGCAAGACGGCGGATTTA |
| PPARA | 123 | Forward: | TCAAGAGCCTGAGGAAACCC |
|  |  | Reverse: | CAAATGATAGCAGCCACAAAGA |
| ACSL1 | 126 | Forward: | ACCAGACCAACCCTATGAATG |
|  |  | Reverse: | GAGCGAAGATGCCGACGAAA |
| LPL | 147 | Forward: | CTCGTGCTCAGATGCCCTAC |
|  |  | Reverse: | GGCAGGGTGAAAGGGATGTT |
| FABP1 | 145 | Forward: | GAACTCATCCAGAAGGGGAAG |
|  |  | Reverse: | TTGACCTTCTCCCCAGTCAG |
| FABP4 | 105 | Forward: | AATTGGGCCAGGAATTTGAT |
|  |  | Reverse: | TCTTTCCATCCCACTTCTGC |
| CD36 | 125 | Forward: | CCATACCCTATTCCTACCAC |
|  |  | Reverse: | AGGCTGCATCTGTACCATTA |
| FATP1 | 117 | Forward: | TTCAACAGCCGCATCCTGC |
|  |  | Reverse: | CTGTGGGCGATCTTCTTGCT |
| FATP4 | 116 | Forward: | GGATGACGTCCTCTATGACTGC |
|  |  | Reverse: | ACTTGACACAATCATCCCAGAA |
| GAPDH | 233 | Forward:  Reverse: | CCACAACATACGTAGCACCACGATC  CCTTCATTGACCTCCACTACATGGT |
| HPRT1 | 131 | Forward: | TCAAGCAGCATAATCCAAAG |
|  |  | Reverse: | AAGGGCATAGCCTACCACAA |

†All porcine gene sequences.
